# Length of patient-physician relationship and patients' satisfaction and preventive service use in the rural south: a cross-sectional telephone study

**DOI:** 10.1186/1471-2296-6-40

**Published:** 2005-10-04

**Authors:** Katrina E Donahue, Evan Ashkin, Donald E Pathman

**Affiliations:** 1 Department of Family Medicine, University of North Carolina at Chapel Hill, Chapel Hill, North Carolina, 27599, USA; 2 Cecil G. Sheps Center for Health Services Research, University of North Carolina at Chapel Hill, Chapel Hill, North Carolina, 27599, USA

## Abstract

**Background:**

Physicians and patients highly value continuity in health care. Continuity can be measured in several ways but few studies have examined the specific association between the duration of the patient-doctor relationship and patient outcomes. This study (1) examines characteristics of rural adults who have had longer relationships with their physicians and (2) assesses if the length of relationship is associated with patients' satisfaction and likelihood of receiving recommended preventive services.

**Methods:**

Cross-sectional telephone survey of health care access indicators of adults in selected non-metropolitan counties of eight U.S. predominantly southern states. Analyses were restricted to adults who see a particular physician for their care and weighted for demographics and county sampling probabilities.

**Results:**

Of 3176 eligible respondents, 10.8% saw the same physician for the past 12 months, 11.8% for the previous 13–24 months, 20.7% for the past 25–60 months and 56.7% for more than 60 months. Compared to persons with one year or less continuity with the same physician, respondents with over five years continuity more often were Caucasian, insured, a high school graduate, and more often reported good to excellent health and an income above $25,000. Compared to those with more than five years of continuity, participants with either less than one year or one to two years of continuity with the same physician were more often not satisfied with their overall health care (OR 2.34; OR 1.78), participants with less than one year continuity were more often not satisfied with the concern shown them by their physician (O.R. 1.90) and having their health questions answered, and those with one to two years continuity were more often not satisfied with the quality of their care (OR 2.37). No significant associations were found between physician continuity and use rates of any of the queried preventive services.

**Conclusion:**

Over half of this rural population has seen the same physician for more than five years. Longer continuity of care was associated with greater patient satisfaction and confidence in one's physician, but not with a greater likelihood of receiving recommended preventive services.

## Background

Both physicians and patients highly value continuity in healthcare [1,2]. The Institute of Medicine (IOM) holds that continuity, defined as an ongoing partnership between patients and physicians, is a central and important component of primary care [3]. Continuity fosters personal relationships and is believed to improve health outcomes, although the latter has been difficult to demonstrate formally.

Continuity has been measured in various ways with different associated outcomes. Concepts of continuity include having a usual place for healthcare, having a usual physician and actually having a greater proportion of one's visits with a particular physician [4-6]. Continuity reportedly increases use rates of some services, including some preventive services, and is associated with lower health care costs for certain populations [7-12]. However, continuity has shown no effect on the use of some particular preventive services, including Pap smears and mammograms [4,13]. Evidence for the relationship between continuity with hospitalization rates is conflicting [4,10,14,15]. Lack of continuity care is associated with fewer follow-up care visits and fewer medical prescriptions given for chronic illness [16]. Few studies, however, have examined outcomes for continuity as defined as having a longer standing relationship with one's physician [9,11]. The outcomes of this notion of continuity are less clearly known.

Elements of trust and satisfaction, important to the ongoing patient-physician relationship, are also important to examine in the context of continuity. Continuity over time allows physicians to get to know and better understand their patients [17] and is associated with greater trust [18]. In older patients, longer relationships are associated with the perception that their provider is more knowledgeable and thorough [11]. Considering these aspects of satisfaction may further the understanding of the benefits of a continuous health care relationship [19,20].

The purpose of this study is to examine the characteristics of rural adults who have longer versus shorter relationships with their current physicians. We also examine if the length of this relationship is associated with aspects of patient satisfaction and with the likelihood that patients have received recommended preventive services.

## Methods

### Study population

Cross-sectional data were obtained from a telephone survey of adults in the rural U.S. Southeast. The survey was conducted as part of an evaluation of the Southern Rural Access Program (SRAP), an initiative to improve access to health care in targeted rural areas of eight U.S. states: Alabama, Arkansas, Georgia, Louisiana, Mississippi, South Carolina, Texas and West Virginia [21]. Professional Research Consultants, Inc. of Omaha, Nebraska (PRC), a survey research firm, fielded the telephone survey with 4,879 adult respondents living in 150 non-metropolitan counties of these eight states from October 2002 to July 2003. PRC administered the survey using a computer-assisted telephone interview (CATI) system with randomly generated numbers within telephone exchanges and active number blocks within each county. Eligible adults included those age 18 and older who had lived in the immediate area for at least 12 months and spoke English or Spanish; a minimum of 10 attempts were made to contact someone at each number. After a number was contacted and confirmed to be a household, a specific adult to be interviewed was randomly selected from among the eligible adults in that household using the next birthday method of identification [22]. The overall participation rate was 51.0% (4879 participants and 4682 refusals). The study protocol was reviewed and exempted by the University of North Carolina School of Medicine's Committee on the Protection of Human Subjects Research.

### Eligibility

Participants were asked, "Is there a place that you usually go to when you are sick or need advice about your health?", if they saw a particular person there for their care and how long that person had been their doctor. Of the 4879 respondents, 4367 (89.5%) reported a usual place of care, 3402 (69.7%) saw a particular person for their care and 3197 (65.5%) stated that person was a doctor.

Subjects' were asked 'How long has this person been your doctor?' and asked to respond in one of four duration categories: previous year (0–12 months), the past one to two years (13–24 months), the past three to five years (25–60 months) and more than 5 years (61 months or more). Of the 3197 eligible subjects, 3176 (99.3%) responded to this question and served as the population for this study.

### Satisfaction questions

Several satisfaction questions were included in the questionnaire based on previous national and regional surveys and a published study [23-25]; responses were provided on five-point Likert scales with a neutral middle option offered. Participants were asked how satisfied they were with their overall health care and with the quality of care they usually received. Participants were also asked how satisfied they were with having their health questions answered during care visits and how welcome and comfortable they were made to feel by the office staff. In addition, participants were asked how satisfied they were with the concern shown for them by their doctor and how confident they were in the abilities of their doctor to help them.

### Preventive service questions

Respondents who reported they had a 'routine medical checkup' in the past year were asked several questions pertaining to the preventive services they had received. They were asked how long it had been since they last had a mammogram, Pap smear, flexible sigmoidoscopy/colonoscopy, influenza vaccine and cholesterol level check. They were also asked if they were counseled about tobacco use (if a smoker), physical activity/exercise and nutrition/diet in the previous 12-months.

Standards of appropriate preventive services were adapted from the conservative recommendations of the US Preventive Services Task Force [26]: (1) mammogram in adult women 50–69 years old in the past year, (2) Pap smear in women 18–64 years old in the past three years, (3) flexible sigmoidoscopy or colonoscopy in people 50 and older ever (at least one), (4) influenza vaccine in people 65 and older in the past year and (5) cholesterol level check in persons 45 years and older in the past five years. Counseling variables included if a doctor or nurse: (1) advised a smoker to quit or stop using tobacco in the past year, (2) had given the participant advice about diet and nutrition in the past year, and (3) had given the participant advice about physical activity or exercise in the past year.

## Analysis

Participants were sorted into four groups based on the number of years they had received care from the same physician: less than a year, one to two years, three to five years and more than five years. Chi-square tests were used to compare the demographic, satisfaction and preventive health care characteristics of each group of years of continuous care to the group with over five-years of care.

Logistic regression was performed to assess the relationship of continuity to the outcomes of satisfaction and preventive service use rates. Dummy variables were constructed for the years subjects had received care from their primary source of care, with more than five years of care as the omitted (comparison) category. Models for each outcome were adjusted for subject age, gender, race, income, health insurance status and self-reported general health status.

For all analyses STATA 8 (College Station, Texas) [27] was used. When respondent demographics were compared against the 2000 U.S. Census data, survey participation rates were found to be lower for males, persons 18–39 years of age, African-Americans, and those with household incomes below $15,000. Analyses were accordingly weighted to adjust for both the over-sampling in small counties and to correct differential response likelihood by demographic groups.

## Results

Among the 3176 persons identifying a particular physician from whom they received their health care, 10.8% (N = 319) had seen the same individual for the past year, 11.8% (N = 369) for the past one to two years, 20.7% (N = 669) for the past three to five years and 56.7% (1819) had seen the same physician for more than five years. Associations between sociodemographic characteristics and duration of care are shown in Table 1. Compared to persons with one year or less continuity with the same physician, respondents with more than five years continuity were more often Caucasian (66.9% versus 61.5%, p = 0.007), more often had insurance (77.3% versus 68.5%, p = 0.001), had more education (43.6% versus 37.5% had at least some college, p = 0.006), more often had an income $25,000 or more (62.4% versus 45.6%, p < 0.001) and more often reported good to excellent health (76.0% versus 66.2%, p < 0.001). Respondents with more than five years continuity also more often had an income higher then $25,000 (62.4%) than the groups reporting only one to two years of continuity (50.1%, p < 0.001) and three to five years continuity (57.6%, p = 0.035). Otherwise, the one to two-year, three to five-year and over five-year continuity groups did not differ in their characteristics.

**Table 1:** Characteristics of subjects who have received care from the same physician for ≤ 1 year, >1 to 2 years, >2 to 5 years, and more than 5 years: Statistical comparisons to group with more than 5 years of continuity

**Patient Characteristics**	**%≤ 1 yr** (0–12 mo) N = 319	**% 1–2 yrs** (13–24 mo) N = 369	**% 3–5 yrs** (25–60 mo) N = 669	**%>5 yrs** (61 mos or more) N = 1819
Age				
18–39	35.8	38.4	36.9	33.0
40–64	42.3	38.3	44.4	44.3
65+	21.9	23.2	18.7	22.7
				
Gender				
Male	42.1	44.5	42.3	45.2
Female	57.9	55.5	57.6	54.8
				
Race/Ethnicity	**			
White, Non Hispanic	61.5	67.5	61.6	66.9
White, Hispanic	3.3	0.5	1.1	1.0
Black	33.5	29.5	33.2	29.3
Other	1.7	2.5	4.1	2.7
				
Insurance	***			
Yes	68.5	72.9	77.7	77.3
No	31.5	27.1	22.2	22.7
				
Education	**			
Less than high school	26.2	17.8	19.4	18.0
High school diploma	29.3	32.7	30.1	30.4
Trade school	7.1	8.1	8.1	8.0
Some College or more	37.5	41.4	42.4	43.6
				
Income	***	***	*	
< $25,000	54.4	50.0	42.4	37.6
≥ $25,000	45.6	50.1	57.6	62.4
				
General health	***			
Good to Excellent	66.2	72.7	73.1	76.0
Fair to Poor	33.8	27.3	26.9	24.2

*P < 0.05 difference for each group with >5 years continuity

**P < 0.01 difference for each group with >5 years continuity

***P < or = 0.001 difference for each group with >5 years continuity

In terms of putative outcomes of continuity, compared to those with more than five years continuity, more respondents with one year or less of continuity reported being neutral or dissatisfied with their overall health care (10.6% versus 4.5%, p < 0.001), with the quality of their health care (6.7% versus 3.3%, p = 0.022) with having their health questions answered (7.2% versus 3.8%, p = 0.021) and with the concerns shown to them by their physicians (7.0% versus 3.4%, p = 0.004) (Table 2). A higher proportion of persons with one year or less continuity were also neutral or not confident in the abilities of their physician to help them than those with more than five years of continuity (20.1% versus 14.5%, p = 0.035). There were no significant differences in rates of any of the preventive services, except those who reported seeing a physician for one year or less were more likely to report being counseled in nutrition over the past year (57.8% versus 48.1%, p = 0.008).

**Table 2:** Association between preventive services and lack of satisfaction with number of years of care from the same physician: Statistical comparisons to group with more than 5 years of continuity

**Outcomes**	**Total Sample ** N = 3176	**%≤ 1 yr** (0–12 mo) N = 319	**% 1–2 yrs** (12–24 mo) N = 369	**% 3–5 yrs** (25–60 mo) N = 669	**%>5 yrs** (61 mos or more) N = 1819
**Lack of Satisfaction**					

% Neutral or dissatisfied with overall health care	N = 3150	10.6***	7.7*	3.8	4.5
% Neutral or dissatisfied with quality of health care	N = 3156	6.7*	7.3**	3.5	3.3
% Neutral or not confident in physician	N = 3156	20.1*	15.4	16.6	14.5
% Neutral or dissatisfied with concern shown by physician	N = 3159	7.0**	4.8	5.3*	3.4
% Neutral or dissatisfied with having questions answered	N = 3161	7.2*	5.3	4.4	3.8
% Felt neutral or not welcome and comfortable by staff	N = 3165	5.6	4.8	3.5	4.3

**Preventive Services **(only asked to those reporting a routine checkup in past year)					

Mammogram within past year in women 50 and older	N = 1234	56.2	61.6	59.7	60.4
Pap smear in women 18–64 years old in past 3 years	N = 1579	89.0	89.3	90.7	88.0
Flu shot in persons 65 and older in past year	N = 720	62.0	64.9	72.0	69.5
Flexible sigmoidoscopy or colonoscopy in persons 50 and older	N = 1756	46.6	52.3	50.5	50.5
Cholesterol in persons 45 and older	N = 2108	87.6	89.9	88.4	88.3
Smoking counseling in past year	N = 634	68.6	73.0	72.9	69.8
Physical activity counseling in past year	N = 2641	56.4	51.2	55.6	51.2
Nutrition counseling in past year	N = 2632	57.8*	47.5	54.6*	48.1

*P < 0.05 difference for each group with >5 years continuity

**P < 0.01 difference for each group with >5 years continuity

***P <0.001 difference for each group with >5 years continuity

Controlling for age, gender, race, income, insurance and health status, participants with one year or less and one to two years of continuity with their physician remained more likely to be neutral or dissatisfied with their overall health care compared to those with more than 5 years of continuity (OR 2.34; 95% CI: 1.39–3.93 and OR 1.78; 95% CI: 1.04–3.06, respectively) (Table 3). Those with one to two years of continuity were also more likely to be neutral or dissatisfied with the quality of health care they usually received (OR 2.37; 95% CI: 1.35–4.15). Those with one year or less continuity were also more likely to be neutral or dissatisfied with the concern shown by their physician (OR 1.90; 95% CI: 1.12–3.23) and having their questions answered (OR 1.98; 95% CI: 1.10–3.57). There were no significant differences across groups with various lengths of relationships with their physicians and their confidence in their physician, feeling welcome by office staff, reported rates of counseling for smoking and physical activity and receipt of preventive services (Table 4). Persons with shorter periods of continuity were more likely, however, to report having received nutrition counseling in the past year (OR 1.47; 95% CI: 1.08–2.02).

**Table 3:** Adjusted relationships between lack of satisfaction and years of care from the same physician: logistic regression results*: Statistical comparisons to group with more than 5 years of continuity

**Lack of Satisfaction**	**%≤1 yr care** OR (95% CI)	**% 1–2 yrs care** OR (95% CI)	**% 3–5 yrs care** OR (95% CI)
% Neutral or dissatisfied with overall health care	2.34	1.78	0.81
	(1.39–3.93)	(1.04–3.06)	(0.50–1.32)
	**p = 0.001**	**p = 0.04**	p = 0.40
% Neutral or dissatisfied with quality of health care	1.87	2.37	1.05
	(0.98–3.60)	(1.35–4.15)	(0.56–1.97)
	p = 0.058	**p = 0.003**	p = 0.87
% Neutral or not confident in physician	1.36	1.07	1.11
	(0.94–1.98)	(0.77–1.50)	(0.83–1.49)
	p = 0.10	p = 0.65	p = 0.47
% Neutral or dissatisfied with concern shown by physician	1.90	1.42	1.37
	(1.12–3.23)	(0.74–2.76)	(0.83–2.27)
	**p = 0.02**	p = 0.29	p = 0.21
% Neutral or dissatisfied with having questions answered	1.98	1.55	1.18
	(1.10–3.57)	(0.81–2.94)	(0.70–2.00)
	**p = 0.02**	p = 0.18	p = 0.52
% Felt neutral or not welcome and comfortable by staff	1.28	1.14	0.77
	(0.66–2.52)	(0.60–2.15)	(0.48–1.23)
	p = 0.46	p = 0.69	p = 0.27

*Adjusted for age, gender, race, income ≥ 25,000, health insurance status, and health status. Statistical comparisons to group with more than 5 years

**Table 4:** Adjusted relationships between receipt of preventive service variables and years of care from the same physician: logistic regression results: Statistical comparisons to group with more than 5 years of continuity

**Preventive services **(only asked to those reporting at least one doctor visit in past year)	**% ≤1 yr care** OR (95% CI)	**% 1–2 yrs care** OR (95% CI)	**% 3–5 yrs care** OR (95% CI)
Mammogram in women 50 and older within past year (n = 1182)	0.94	1.17	0.96
	(0.60–1.46)	(0.77–1.80)	(0.70–1.32)
	p = 0.78	p = 0.44	p = 0.81
Pap smear in women 18–64 years old in past 3 years (n = 1543)	1.27	1.17	1.36
	(0.71–2.24)	(0.62–2.20)	(0.83–2.24)
	p = 0.41	p = 0.63	p = 0.22
Flu shot in persons 65 and older in past year (n = 679)	0.66	0.83	1.07
	(0.36–1.20)	(0.43–1.61)	(0.66–1.74)
	p = 0.17	p = 0.58	p = 0.77
Flexible sigmoidoscopy or colonoscopy in persons 50 and older 10 years (n = 1684)	0.87	1.15	1.05
	(0.60–1.96)	(0.83–1.59)	(0.81–1.37)
	p = 0.53	p = 0.39	p = 0.67
Cholesterol in persons 45 and older (n = 2024)	1.08	1.33	0.98
	(0.59–1.89)	(0.77–2.31)	(0.68–1.42)
	p = 0.79	p = 0.30	p = 0.93
Smoking counseling in past year (n = 611 smokers)	1.00	1.15	1.31
	(0.55–1.82)	(0.61–2.17)	(0.74–2.33)
	p = 0.99	p = 0.66	p = 0.36
Physical Activity counseling in past year (n = 2548)	1.23	1.06	1.16
	(0.94–1.64)	(0.78–1.43)	(0.92–1.47)
	p = 0.13	p = 0.70	p = 0.19
Nutrition counseling in past year (n = 2541)	1.47	1.05	1.25
	(1.08–2.02)	(0.76–1.45)	(1.01–1.54)
	**p = 0.01**	p = 0.76	p = 0.04

*Adjusted for age, gender, race, income ≥ 25,000, health insurance status, and health status. Statistical comparisons to group with more than 5 years

## Discussion

Continuity can be measured in a number of ways. When measured as the length of the patient-physician relationship, we find continuity varies with patient demographics and with elements of satisfaction. Over half of adults of this study's 150 rural communities report seeing the same physician for more than five years. Those who saw the same physician for less than five years tended to be nonwhite, without health insurance, less educated, more often report income of less than $25,000 and more often report fair to poor health status. This group was also less likely to be satisfied with their overall health care and its quality and with components of the doctor-patient relationship.

Patients and their physicians value continuity [28-31]; in this study of rural participants, satisfaction appears associated with length of the continuity relationship, as similarly noted in other studies [11,18,32]. The elements of greater satisfaction found in persons with longer relationships with their physicians lend credence to the importance of the continuity relationship to patient outcomes. However, in this study, there appears to be a threshold effect at one or two years of continuity beyond which satisfaction does not rise significantly further.

Even a relationship length of two years is becoming difficult to maintain in the current U.S. healthcare system, especially in urban areas, with the pressures of competitive managed care plans which encourage patients and their employers to change health plans, and the growth of urgent care centers [33]. In the 1996–97 Community Tracking Study household survey, 17% of privately insured persons changed their health plan during the year prior to the survey [34]. Of those changing health plans, a little over half cited changes in their insurance as the reason for also changing their source of care. Increases in insurance premiums could contribute to further health provider switching.

Satisfaction, as an indicator of quality of care [28], has been found to affect other outcomes, including patient adherence to their physicians' recommendations [35]. Mothers are more likely to follow a physician's treatment recommendations for their child if she feels the physician is friendly and understands the complaint [36]. Patients with hypertension are more likely to adhere to treatment and have their blood pressure under control when the physician considers the patient an active participant in treatment [37].

Previous studies note the importance of having a usual source of care versus no source to the timely receipt of preventive services for younger adults [38,39]. However no differences in preventive care services were observed for older Americans in long-term relationships [9]. In our study preventive services outcomes did not differ significantly by length of continuity as well. For preventive services, it appears that having a usual source of care is important but no additional benefit comes with having a longer-term relationship with that source of care.

Respondents with one year or less continuity, interestingly, were more likely to report receiving nutrition counseling than those in the five-year or more continuity category. Possibly more patients are asking about nutrition issues in the first year or are specifically changing doctors to discuss nutrition. Another possibility is that the developing familiarity with the patient in a long term relationship may be associated with less vigilance by the physician or less counseling in areas in which the patient may have initially shown resistance [40].

## Limitations

The cross-sectional design of this study limits our ability to attribute causation to the statistical relationships demonstrated or know the directions of any causal connections. We do not know if continuity results in higher trust and satisfaction or if the opposite is true. Our data are limited in that the outcomes of having received counseling and other preventive services were self-reported and may not accurately reflect care patients received. Telephone surveys also limit the population to those persons with a working phone, although weighting upwards for households with low incomes and minorities partially adjusts for this. The survey response rate of 51% was moderate but similar to other U.S. national telephone surveys [41]. Response bias is a possibility. This study addressed a rural population statistically representative of the U.S. rural South, thus its findings may not apply to urban and other regions of the U.S. or to other countries. However, we know of no reason to expect that the association between longer-term doctor-patient relationships and satisfaction differs elsewhere.

## Conclusion

Over half of this rural population has seen the same physician for more than five years. Longer continuity was significantly related to aspects of the patient-physician relationship, specifically people's satisfaction with and confidence in their physicians, but not with one's likelihood of receiving recommended preventive services. Fostering long-term relationships between patients and their physicians may help promote the outcome of greater patient satisfaction with care.

## Competing interests

The author(s) declare that they have no competing interests.

## Authors' contributions

KD conceived and designed the study, performed the analyses, and drafted the manuscript. EA participated in the design, interpretation and helped draft the manuscript. DP participated in the design, analyses and critical review of the manuscript. All authors read and approved the final manuscript.

## Pre-publication history

The pre-publication history for this paper can be accessed here:


